# Are glucose and insulin levels at all time points during OGTT a reliable marker of diabetes mellitus risk in pediatric obesity?

**DOI:** 10.1007/s40618-023-02030-6

**Published:** 2023-02-10

**Authors:** A. La Valle, G. d’Annunzio, C. Campanello, G. Tantari, A. Pistorio, F. Napoli, G. Patti, M. Crocco, M. Bassi, N. Minuto, G. Piccolo, M. Maghnie

**Affiliations:** 1 Pediatric Clinic and Endocrinology Unit, IRCCS Istituto Giannina Gaslini, Genoa, Italy; 2 Epidemiology and Biostatistics Department, IRCCS Istituto Giannina Gaslini, Genoa, Italy; 3 Department of Neuroscience, Rehabilitation, Ophthalmology, Genetics, Maternal and Child Health, University of Genoa, Genoa, Italy; 4 Gastroenterology Unit, IRCCS Istituto Giannina Gaslini, Genoa, Italy; 5 Neurooncology Unit, IRCCS Istituto Giannina Gaslini, Via G. Gaslini 5, 16147 Genoa, Italy

**Keywords:** Insulin resistance, OGTT, HOMA-IR, Type 2 diabetes mellitus, Pediatric obesity

## Abstract

**Purpose:**

Childhood overweight and obesity associated with insulin resistance and metabolic syndrome represent the new global pandemic and the main causative factors for dysglycemia, prediabetes, and Type 2 Diabetes Mellitus (T2DM). Predictors, such as HOMA-IR, HOMA-β%, and QUICKI lack specific reference values in children. OGTT is a gold standard for glycometabolic assessment. Recently, a glycemic level higher than 155 mg/dl at + 60′ after glucose ingestion has been defined as a risk factor for T2DM in obese adolescents. We aim to analyze and correlate fasting insulin-resistance markers with OGTT results in overweight/obese children and adolescents.

**Methods:**

We retrospectively evaluated glucose and insulin values during a 2-h OGTT every 30 min in 236 overweight/obese patients. Glucose values and insulin sum during OGTT were compared to glycometabolic indexes and different cut-off values for insulin sum.

**Results:**

A 1-h glucose > 155 mg/dl and insulin sum > 535 microU/ml at all times during OGTT are the best predictors of diabetes risk in obese youths. A1-h glucose > 155 mg/dl is significantly associated with HbA1c > 5.7%, while no association was observed between HbA1c > 5.7% and glucose levels at baseline and 2 h. The ability of the standardized HOMA-IR to predict the prediabetes status is clearly lower than the total insulin sum at OGTT.

**Conclusion:**

Our study demonstrates that also 1-h post-OGTT glucose, together with HbA1c, is an effective diabetes predictor.

## Introduction

Childhood overweight and obesity represent a new other global pandemic, and all industrialized countries are severely suffering from this public health problem [1].

It has been reported that obesity affects approximately 107.7 million children and adolescents worldwide [1]. In Italy, the prevalence of overweight and obesity is 22.5% and 9.3%, respectively [2], and a dramatic increase within the next years is predicted [3].

This rising prevalence of obesity in the young age group is a consequence of a sedentary lifestyle as part of globalization and industrialization affecting all societies [3]. Pediatric obesity is also related to different socioeconomic status, with the greatest risk reported in the poorest families [4].

Childhood overweight and obesity are the most important risk factors for several diseases, including Metabolic Syndrome (MS), Obstructive Sleep Apnea Syndrome (OSAS), Type 2 Diabetes Mellitus (T2DM), and vascular complications, starting with subclinical endothelial damage up to clinical vascular disease [5, 6], increasing morbidity, and mortality even at a young age [7, 8].

It has been reported that childhood obesity and BMI trajectories from childhood to early midlife predict endothelial dysfunction [8].

Prompt diagnosis of overweight and obesity is mandatory for pediatricians to clearly define the clinical and laboratory characteristics, assess a multidisciplinary care program, and screen and prevent the development of related complications [9, 10].

Overweight and obesity are characterized by altered metabolic status, including Insulin Resistance (IR), different degrees of dysglycemia (i.e., fasting hyperglycemia and impaired glucose tolerance), and abnormal lipid profile [11, 12].

Even if in pediatric patients clinical T2DM is rarely encountered, other conditions associated with IR like MS are frequently observed in adolescence (a cluster of obesity, hypertension, dyslipidemia, dysglycemia, and IR) [13, 14].

IR is characterized by a reduced ability of insulin to stimulate glucose intake by adipose tissue and muscles, together with reduced insulin capability to suppress hepatic glucose synthesis and output [15]. The subsequent excessive supply of free fatty acids further affects glucose transportation in the skeletal muscles and inhibits insulin activity [16, 17]. As the process persists, glucotoxicity can occur, leading to chronic hyperglycemia and clinical T2DM [7]. T2DM in youth was almost undiagnosed until 2 decades ago, being described only in grossly obese siblings of patients with diabetes related to genetic syndromes or belonging to ethnic minorities like Pima Indians [18]. Obesity and insulin resistance are recognized as the most important causative factors for the early development of this disease.

The role of IR as an independent predictor of a range of disorders is certain; however, its quantitative assessment is not regularly performed in routine clinical practice, despite several methods have been proposed [19, 20].

Among the markers of insulin resistance, several indexes describing glucose-insulin homeostasis by means of simple, mathematically derived equations have been proposed [20].

Homeostatic Model Assessment (HOMA) of IR (HOMA-IR), of β-cell activity (HOMA-β%) and insulin sensitivity (QUICKI) have been developed, even on fasting samples of plasma glucose and insulin, making the evaluation of these parameters easier and reproducible also for follow-up studies [20]. These methods measure insulin resistance, β-cell insulin production, and insulin sensitivity, respectively, and are useful tools to assess metabolic status.

Glycometabolic assessment can also be evaluated by Oral Glucose Tolerance Test (OGTT), a procedure developed more than 100 years ago [21]. Baseline fasting plasma glucose and glycemic levels are measured every 30′ after glucose ingestion. Glucose levels at + 120′ define normal glucose tolerance, impaired glucose tolerance, and diabetes mellitus [17]. Recently, a glycemic level higher than 155 mg/dl at + 60′ after glucose load has been defined as a risk factor for T2DM in obese adolescents [22]. Moreover, total insulin obtained by sum during all the times of the test is useful to define insulin resistance [23].

HbA1c measurement has been included as a diagnostic marker of diabetes mellitus. Values between 5.7 and 6.4% identify prediabetes, while values higher than 6.5% are diagnostic for diabetes [24]. HbA1c represents integrated glucose levels over the previous 2–3 months, is convenient, does not require fasting, and is highly reproducible and globally standardized. On the other hand, it is less sensitive than fasting plasma glucose and 2-h post-OGTT glucose, and its accuracy can be impaired by hemoglobin variants, red cell turnover, kidney disease, age, and race.

At present, conflicting data are reported about the prevalence of impaired fasting glucose, insulin resistance, and abnormal glucose metabolism in obese subjects. The lack of uniformity seems attributable to ethnic differences among the considered groups.

### Aims

The aim of our study was to assess the prevalence of insulin resistance, abnormality of glucose metabolism, and lipid profile, correlating OGTT and insulin-resistance indexes in young obese patients at IRCCS Giannina Gaslini Children Hospital, Genoa, Italy.

## Patients and methods

In our cross-sectional retrospective study, we evaluated OGTT data and other biochemical parameters in 236 obese children and adolescents (107 m and 129 f), with a median age of 13.2 years (range 10.8–15.5 years) and followed at the outpatient clinic, Department of Pediatrics, Gaslini Institute, Genoa, Italy, between 2016 and 2020. Patients with syndromic obesity or intercurrent illnesses were not considered. Inclusion criteria were: test performed 3 days after an unrestricted diet, normal physical activity, and absence of acute illnesses or administration of drugs affecting glucose metabolism.

In all patients, height, weight, body mass index, and pubertal stage according to Tanner were recorded. Measurements were performed with the subject wearing only light indoor clothing and no shoes. Height was measured with a portable Harpender stadiometer by Tanner technique. Weight was measured with a standardized portable scale. BMI was calculated as follows: (weight in Kg)/(height in meters)^2^. According to the WHO criteria for children aged between 5 and 19 years, overweight was defined as BMI-for-age > 1 SDS and obesity as BMI-for-age > 2 SDS above the 2007 WHO Growth Reference median [25, 26]. Severe obesity was defined as BMI-for-age above + 3 Z-scores relative to the 2007 WHO growth reference median [27].

BMI was calculated and BMI-SDS score (BMI-SDS) was computed for each subject using the formula BMI-SDS = (actual BMI – mean BMI-for-age and sex)/BMI-SDS for age, race, and gender, based on established standards and norms. Pubertal development stages were assessed using Tanner staging criteria by well-trained physicians in pediatric endocrinology.

### Biochemical analyses

After 12 h of overnight fasting, all subjects underwent baseline diagnostic blood sample withdrawals including fasting Plasma Glucose (PG), HbA1c, insulin, triglycerides, and total cholesterol levels. Glucose was detected by the glucose oxidase method on venous whole blood, and results were modified into plasma glucose values. Insulin was measured with a radioimmunoassay method. All parameters were measured at the same laboratory.

As estimates of insulin resistance, we detected HOMA-IR using the following formula: [fasting plasma insulin in microU/ml x fasting plasma glucose (FPG) in mmol/l]/22.5, and QUICKI as 1/(log_10_ fasting plasma insulin in microU/ml + log_10_ glucose in mmol/l) [20]. As an index of pancreatic beta-cell function, we measured HOMA-β% as (20 fasting plasma insulin in μU/ml)/(FPG in mmol/l – 3.5) [20]. Hyperinsulinism was defined as the sum of insulin levels at 0th, 30th, 60th, 90th, and 120th min during OGTT > 300 microU/ml [23].

OGTT was performed using a standard dose of 1.75 g of glucose/kg of body weight (max 75 g) according to the American Diabetes Association guidelines [28, 29]. Before starting OGTT, an intravenous line was placed in the upper limb, and a fasting blood sample (after 10 to 12 h of fasting) was taken and recorded as T0 (T for time). Blood samples were withdrawn on the 0th, 30th, 60th, 90th, and 120th min, and results were evaluated according to ADA criteria [28].

After the load, glucose tolerance was defined using standard parameters, that is

- Normal Glucose Tolerance (NGT) = PG < 140 mg/dl at 2 h OGTT,
- Impaired Glucose Tolerance (IGT) = PG 140–199 mg/dl,
- and Diabetes Mellitus (DM) = PG ≥ 200 mg/dl [28].

We also considered 1-h PG > 155 mg/dl as a biomarker to define high risk for progression to diabetes mellitus at a stage when β-cell function is substantially intact [22].

Biochemical parameters were evaluated, defining the so-called prediabetes as:

- Impaired Fasting Glucose (IFG), i.e., FPG 100–126 mg/dl,
- or IGT post-OGTT, i.e., PG 140–200 mg/dl, or HbA1c 5.7–6.4% (endorsed by ADA for prediabetes diagnosis) [28].

## Statistical methods

Descriptive statistics were performed; categorical variables were reported in terms of absolute frequencies and percentages; quantitative variables were reported in terms of median values and first and third quartiles (1st–3rd q).

Body Mass Index (BMI) was calculated as the ratio of body weight (kg) to squared height (meters). BMI was standardized by the LMS method, with gender and age adjustments, and was expressed as z-score, using the WHO 2007 tables as standard reference [30].

Comparison of frequencies was done utilizing the Chi-square test or Fisher’s exact test (in case of expected frequencies < 5).

Correlation between quantitative parameters (e.g., HOMA-IR vs Total Insulin after OGTT) has been evaluated by means of Spearman’s Rank order correlation coefficient (*r*_S_). The correlation coefficient was considered as follows: *r*_S_ <|0.4| weak, ≥|0.4| to |0.59| moderate, ≥|0.6| to |0.79| strong, and ≥|0.8| very strong, according to Swinscow TVD (1997) [31]^.^

ROC curve analysis has been used to find the best cut-off values for different variables that were identified as possible predictors of glucose tolerance, defined as normal NGT, IGT, and DM [32].

Finally, to evaluate the role of different independent variables in predicting the outcome, a multivariable logistic regression model has been performed; the outcome variable was glucose intolerance (yes, coded “1”/no, coded “0”). Clinically relevant or statistically significant variables evaluated at bivariate analysis were included in the model: gender (female vs male), age at OGTT (years), Tanner’s Stage (post-pubertal, pubertal, and pre-pubertal), BMI z-score, Total Insulin at OGTT (≥ 535 vs < 535 microU/mL), and HOMA-IR percentile (≥ 99.2 vs < 99.2). Some quantitative variables (e.g., Total Insulin at OGTT) were dichotomized on the basis of the best cut-off value obtained using the ROC curve method (and considering glucose intolerance as the outcome). The Odds Ratios (ORs) with 95% Confidence intervals (95% CI) have been calculated and reported. The Log-Likelihood Ratio test (LR test) has been used for testing statistical significance. The backward approach (which consists in removing non-significant variables from the saturated model) has been used for evaluating the model. The area under ROC curve of the model has been used as an indicator of goodness of fit.

All the statistical tests were two-sided and a *P* value < 0.05 was considered statistically significant. “Statistica” (release 9.1, StatSoft Corporation, Tulsa, OK, USA) was used for all the bivariate analyses; MedCalc was used for the ROC curve analysis; “Stata” (release 7.0, College Station, TX, USA) was used for the Fisher’s exact test and for the multivariable logistic regression model.

## Results

A description of the enrolled patients (*n* = 236) is reported in Table 1. Patients of both genders with a median age at evaluation of 13.2 years were included in the study. Only overweight, obese, and severely obese patients (as defined in the method section) were included in this cohort and the distribution of these categories of weight is reported in Table 1. Among these patients, only a minority was in a pre-pubertal Tanner stage (19.5%).

**Table 1:** Description of the study cohort (*n* = 236)

	*n*. (%)
Male	107/236 (45.3%)
Female	129/236 (54.7%)
Median age at OGTT (years) (1st–3rd q)	13.2 (10.8–15.5)
Tanner’s stage	
Tanner I	45/231 (19.5%)
Tanner II–IV	102/231 (44.2%)
Tanner V	84/231 (36.4%)
Race	
Caucasian	202/236 (85.6%)
Non-caucasian	34/236 (14.4%)
BMI z-score, median values (1st–3rd q)	2.7 (2.2–3.1)
BMI percentile, median values (1st–3rd q)	99.6 (98.5–99.9)
Weight category	
Overweight	49/236 (20.8%)
Obese	111/236 (47%)
Severe obesity	76/236 (32.2%)

BMI z-score and BMI percentile: z-scores and percentiles were calculated according to Cole LMS method and WHO 2007 reference standard

As shown in Table 2, only a PG at 60 min ≥ 155 mg/dL is statistically associated with an HbA1c ≥ 5.7% (*P* = 0.011); on the contrary, no association was observed at baseline and at 120 min glucose levels, between impaired levels of BGL and HbA1c ≥ 5.7%.

**Table 2:** Association between Blood Glucose Level (BGL, mg/dL) and Glycated Hemoglobin (HbA1c) (%) (*n* = 236)

	HbA1c < 5.7	HbA1c ≥ 5.7	^§^*P*
BGL t0			
< 100	186/193 (96.4%)	7/193 (3.6%)	0.64
≥ 100 and < 126	36/38 (94.7%)	2/38 (5.3%)	
BGL t60 minutes			
< 155	168/171 (98.2%)	3/171 (1.8%)	0.011
≥ 155	54/60 (90%)	6/60 (10.0%)	
BGL t120 minutes			
< 140	173/179 (96.6%)	6/179 (3.4%)	0.46
≥ 140 and < 200	47/50 (94%)	3/50 (6%)	
≥ 200	2/2 (100%)	0/2 (0%)	

^§^*P* value refers to the Fisher’s exact test

As observed in Table 3, the literature cut-off value of total insulin after OGTT > 300 microU/mL with respect to prediabetes status has been demonstrated to have very low specificity (19.6%), meaning that the rate of false positives is too high.

**Table 3:** Sensitivity, specificity, and Positive and Negative Predictive values (PPV/NPV) of different predictors of variables concerning prediabetes

	Risk of diabetes				
	Yes	No	Total	Sens/Spec	PPV/NPV
Insulin sum at OGTT > 300 microU/mL	74	127	202	0.987/0.196	0.368/0.969
≤ 300 microU/mL	1	31	32		
Total	75	158	233		
Insulin sum at OGTT > 535 microU/mL	63	70	133	0.840/0.557	0.474/0.880
≤ 535 microU/mL	12	88	100		
Total	75	158	233		
Insulin sum at OGTT > 990 microU/mL	26	16	42	0.347/0.900	0.619/0.744
≤ 990 microU/mL	49	142	191		
Total	75	158	233		
Insulin sum at OGTT > 1284 microU/mL	17	8	25	0.227/0.949	0.680/0.721
≤ 1284 microU/mL	58	150	208		
Total	75	158	233		
Standardized HOMA-IR > 75pct	56	100	156	0.747/0.371	0.359/0.756
Standardized HOMA-IR ≤ 75 pct	19	59	78		
Total	75	159	234		
Standardized HOMA-IR > 99.2 pct	29	37	66	0.387/0.767	0.439/0.726
Standardized HOMA-IR ≤ 99.2 pct	46	122	168		
Total	75	159	234		

Risk of diabetes: BGL at 60 min > 155 mg/dl or BGL at 120 min ≥ 140 and < 200 mg/dL

*Sens/Spec* sensitivity/specificity, *PPV* and *NPV* positive and negative predictive value

On the contrary, the cut-off value of Total Insulin Sum (TIS) after OGTT > 535 microU/mL, statistically obtained utilizing the ROC curve analysis provided a much higher specificity (55.7%), while the cut-off value of total insulin after OGTT > 990 microU/mL offers a better specificity (90%) at the cost of a drop of sensitivity (34.7%).

Finally, a specificity up to 94.9% was reached using a cut-off value for total insulin after OGTT > 1284 microU/mL.

The best cut-off value for Standardized HOMA-IR (obtained through the ROC curve) was > 99.2 percentile, showing specificity of 76.7%, otherwise a low sensitivity (38.7%).

**Fig. 1:**
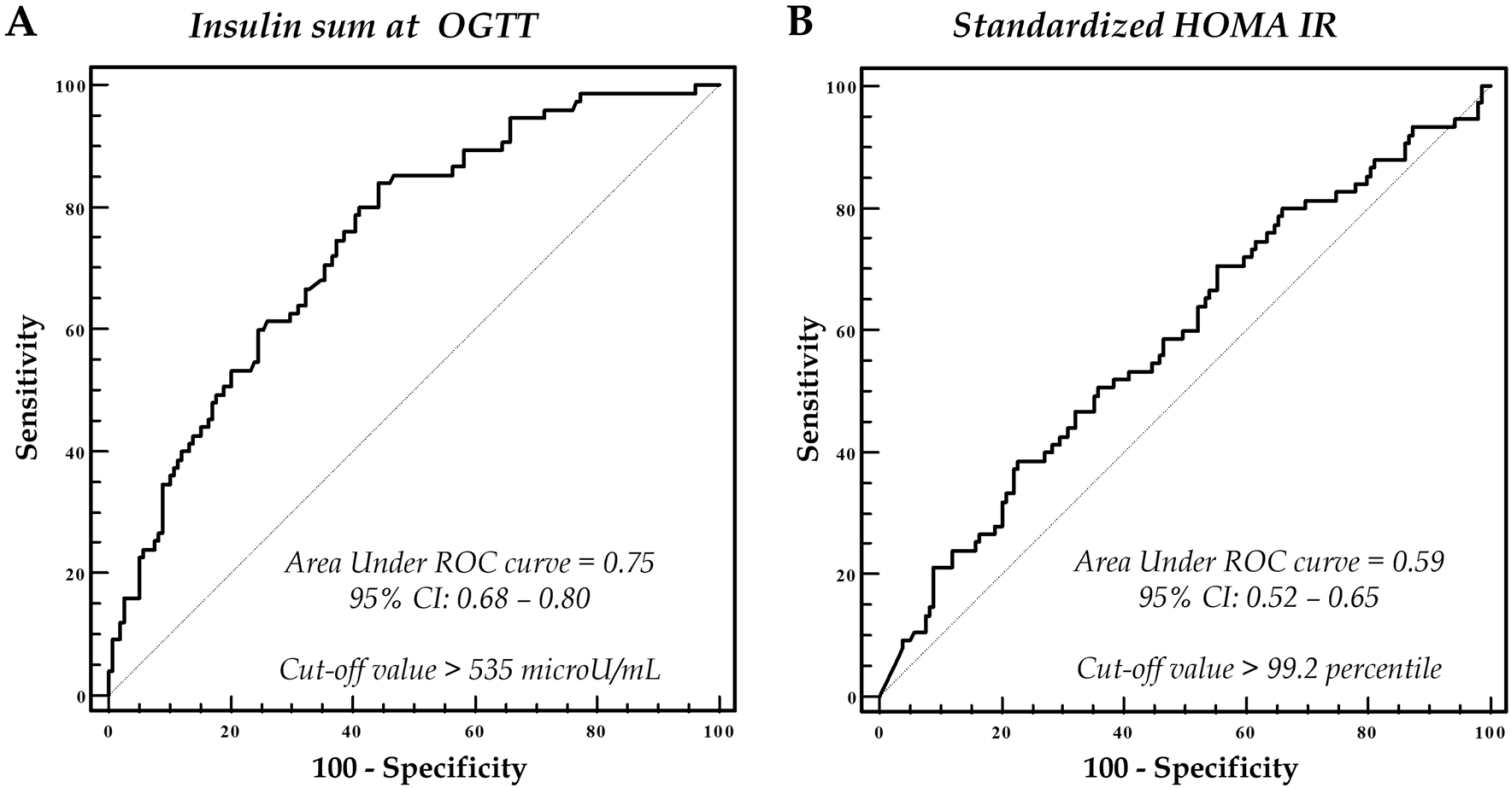
The Area Under the ROC Curve demonstrates how the ability of total insulin sum at OGTT to predict the prediabetes status (**A**) is clearly higher than using the standardized HOMA-IR (**B**)

As shown in Fig. 1, the ability of the standardized HOMA-IR to predict the prediabetes status is lower than the ability of the TIS sum at OGTT, as demonstrated by the Area Under the ROC Curve (AUC = 0.59, panel B vs AUC = 0.75, panel A).

**Fig. 2:**
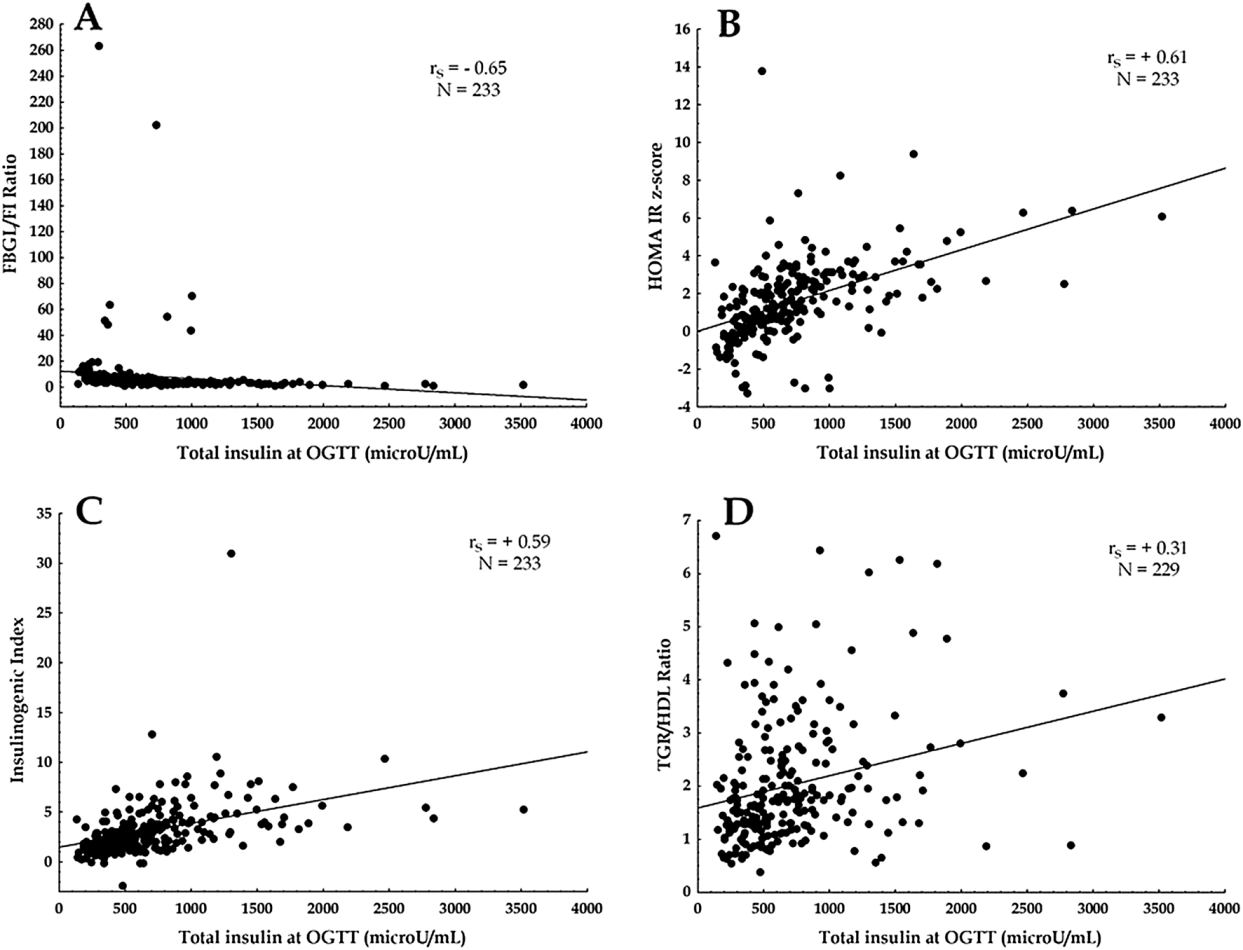
Correlation between Total Insulin after OGTT and Fasting Blood Glucose Level (mg/dL)/Fasting Insulin (microU/mL) (FBGL/FI Ratio) (**A**); correlation between Total Insulin after OGTT and HOMA-IR z-score (**B**); correlation between Total Insulin after OGTT and Insulinogenic Index (δ Insulin (0–30 min)/δ Blood Glucose Level (0–30 min) (**C**); correlation between Total Insulin after OGTT and Triglycerides/High-Density Lipoproteins Ratio (TG/HDL Ratio) (**D**)

As shown in Fig. 2, a strong inverse correlation was observed between TIS after OGTT and Glucose/Insulin Ratio at baseline (*r*_s_ =  − 0.65) (Fig. 2A), and a strong positive correlation was observed between TI after OGTT and HOMA-IR z-score (*r*_s_ =  + 0.61) (Fig. 2B) and HOMA-IR (*r*_s_ =  + 0.64)(not shown); a moderate positive correlation was observed between TIS after OGTT and Insulinogenic Index (*r*_s_ =  + 0.59) (Fig. 2C). No correlation was observed between TI after OGTT and TG/HDL ratio (Fig. 2D).

Finally, a multiple logistic regression model was fitted to evaluate the role of some demographic and clinical variables in predicting the risk of glucose intolerance (Table 4).

**Table 4:** Logistic regression models; outcome: glucose intolerance (75/233; 32.2%)

Predictors	OR_Adj_	95% CI	*P*
Best-fitted regression model			
Total insulin ≥ 535microU/mL (reference: < 535)	7.05	3.43–14.47	< 0.0001
Age at OGTT (years)	1.10	0.996–1.22	0.055
ROC curve of the model = 0.75			
Model A			
Total insulin ≥ 535microU/mL (reference: < 535)	8.19	3.60–18.59	< 0.0001
Age at OGTT (years)	1.10	0.995–1.21	0.059
Homa-IR percentile > 75th	0.60	0.27–1.34	0.21
ROC curve of the model = 0.75			
Model B			
Total insulin ≥ 535 microU/mL (reference: < 535)	6.66	3.16–14.01	< 0.0001
Age at OGTT (years)	1.10	0.999–1.22	0.048
Homa-IR percentile > 99.2	0.90	0.45–1.77	0.76
ROC curve of the model = 0.74			

*OR* odds ratio, *95% CI* 95% confidence interval, *P P* value refers to the likelihood-ratio test

The following parameters were included in the model: gender (female vs male), age at OGTT (years), Tanner’s Stage (adult and pubertal vs pre-pubertal), BMI z-score, TIS at OGTT (≥ 535 vs < 535 microU/ml), and HOMA-IR percentile (≥ 99.2 vs < 99.2).

In the logistic regression model for the association with glucose intolerance gender (*P* = 0.43), Tanner’s stage (*P* = 0.32), BMI z-score (*P* = 0.57), and HOMA-IR percentile (*P* = 0.86) turned out to be non-statistically significant. Age at OGTT (expressed in years) (*P* = 0.055) was not statistically significant too, but it was forced to remain in the model to correct the role of TIS at OGTT for the contribution of age. The only variable that was statistically associated with a risk of glucose intolerance was TIS at OGTT (≥ 535 microU/mL) (OR = 7.05; 95% CI 3.43–14.47; *P* < 0.0001). The AUC curve of the best-fitted regression model (including insulin at OGTT and age) was equal to 0.75.

To further investigate the role of the variable HOMA-IR using 2 different cut-off values, the 2 different logistic regression models were fitted (Table 4). As shown in Table 4, HOMA-IR turned out to be not statistically significant either using the more common cut-off value (> 75th percentile, Table 4 Model A) (*P* = 0.21) or using the cut-off value obtained through the ROC curve method (> 99.2 percentile; Table 4 Model B) (*P* = 0.76).

## Discussion

The main result of our study is that 1-h glucose > 155 mg/dl and IGT together with high insulin levels during OGTT were the best predictors of diabetes risk. Therefore, OGTT with glucose and insulin measurement at 0th, 30th, 60th, 90th, and 120th min is the best means to evaluate glycometabolic assessment and risk profile in obese youths. Noteworthy, 1-h glucose > 155 mg/dl deserves attention as a biomarker.

Our question is: do pediatricians need risk markers of diabetes mellitus in obese children and adolescents?

It is recognized that the obesity pandemic, especially in the pediatric age, deserves prevention strategies aimed at precocious diagnosis and avoiding the progression toward T2DM and its related complications in young adulthood [6–8]. At present, clinical T2DM is less frequent in early adolescence as compared to the growing rate of obesity, but the disease can develop over a relatively short period [7]. T2DM especially when diagnosed in young adults increases morbidity and mortality and impairs quality of life [7]. Therefore, the availability of reproducible, sensitive, inexpensive, feasible, and adaptable markers to better classify subjects at risk of T2DM and to assess prevention strategies is mandatory.

Diabetes screening tests include FPG, OGTT, and HbA1c. Briefly, FPG can be performed as a single blood sample, requires overnight fasting, and is less sensitive as compared to OGTT. In fact, FPG alone for diabetes diagnosis identifies patients too late [21].

OGTT has been widely recognized as a sensitive tool to assess glucose metabolism. It is recommended in case of borderline glucose values during screening or non-fasting, HbA1c inaccuracy or unreliability (hemoglobinopathies, iron deficiency, and anemia), renal glycosuria, and for screening procedures [21].

As regards the reproducibility of OGTT in obese adolescents, it has been reported that its repetitiveness is mandatory for patients with IFG or IGT, while only in the case of OGTT response compatible with T2DM, a second test is not needed [33]. Unfortunately, being our case series collected before 2021, a single OGTT was performed.

Prior to diabetes mellitus development, glucose levels during OGTT increase to impaired glucose tolerance (IGT), which reflects higher peripheral insulin resistance, normal hepatic insulin sensitivity, progressive β-cell impairment, and reduced secretion of insulinotropic hormones [34–37].

On the other hand, normal glucose tolerance during OGTT does not seem to be a protective factor against the future development of diabetes. Recently, it has been reported that up to 40% of subjects who progress toward T2DM over a 5-year follow-up had NGT at first evaluation [38].

Current prediabetes criteria based on 2-h glucose levels identify affected patients too late and miss the opportunity to adopt prevention strategies [39]. Noteworthy, it has been demonstrated that 30′ and 60′ glucose levels during OGTT showed higher predictive value. Therefore, intermediate points during OGTT, especially 1-h PG, should be collected [33].

The first Italian report of 1-h OGTT in obese youth was retrospectively performed by Fintini et al. based on 1038 OGTTs. They reported 1-h glucose > 155 mg/dl in 12% of patients with normal glucose tolerance and in 57% of patients with IGT, suggesting the importance of 1-h glucose evaluation [22].

Similarly, Jagannathan et al. evaluated OGTT in the so-called pre-prediabetes stage, identifying 1-h plasma glucose > 155 mg/dl as a reliable biomarker for subclinical organ damage [33].

Khokhar et al. evaluated the role of HbA1c compared to OGTT in 230 obese children and adolescents as a diagnostic tool for prediabetes diagnosis. They defined HbA1c alone as a poor discriminator of prediabetes while BMI-Z score, HbA1c, and HOMA-IR taken together show a better prognostic value [40]. In our case series, HbA1c showed a predictive value together with during OGTT both 1-h glucose > 155 mg/dl and TIS > 535 microU/ml.

Kim et al. evaluated the correlation between plasma glucose and HbA1c and the accuracy of HbA1c as a diagnostic tool to identify diabetes mellitus [41]. HbA1c levels and 2-h post-OGTT glucose have higher diagnostic sensitivity than fasting plasma glucose. The optimal HbA1c level as a predictor of diabetes in Korean pediatric patients was 6.15%.

Our results showed that a BGL > 155 mg/dl during OGTT is significantly associated with HbA1c > 5.7%, while no association was observed at baseline and 120 min, between impaired levels of BGL and HbA1c ≥ 5.7%.

It is known that prediabetes according to 2-h OGTT is associated with a more severe clinical prognosis and increased risk of complications [42]. On the other hand, HbA1c alone is less accurate as a predictor of diabetes than 2-h post-OGTT glucose. Our data demonstrate that also 1-h post-OGTT glucose, prior than 2-h post-OGTT glucose values, together with HbA1c are effective predictors.

As regards the correlation between OGTT and HbA1c in the pediatric population, Chan et al. evaluated FPG, 2-h post-OGTT glucose, and HbA1c levels in obese and prediabetic youths [43]. In this cohort of patients, HbA1c and 2-h post-OGTT glucose equally performed as predictors of free-living glycemia on continuous glucose monitoring.

Insulin resistance is an important factor related to cardiovascular disease, metabolic syndrome, and T2DM [13]. We observed that the ability of the standardized HOMA-IR to predict the prediabetes status is lower than the ability of the total insulin sum at OGTT, again suggesting performing OGTT at 0th, 30th, 60th, 90th, and 120th min. On the other hand, a strong inverse correlation between total insulin after OGTT and Glucose/Insulin Ratio and HOMA-IR z-score was observed. Moreover, a moderate positive correlation was found between TIS after OGTT and Insulinogenic Index, while no correlation was observed between TIS after OGTT and TG/HDL Ratio.

The IR parameters vary for different ages, gender, and ethnic group; therefore, specific reference values for such groups should be available to provide reliable biomarkers [44]. In young Caucasian children and adolescents, percentiles of HOMA-IR varied significantly depending on gender, age, and BMI-SDS. Our previous report showed different HOMA-IR, HOMA β%, and QUICKI in a selected group of children and adolescents with normal BMI-SDS, FPG, and negative family history of dysglycemia [45]. In particular, subjects belonging to Tanner stage 2–4 had significantly higher levels, due to increased physiological insulin resistance related to sex steroids and growth hormone secretion.

## Conclusion

Our study demonstrates that also 1-h post-OGTT glucose, together with HbA1c, is an effective diabetes predictor. Among its strengths, it has to be considered that data collection and analysis were conducted by a well-trained staff, following a standardized protocol, and that the assay of glucose and insulin were performed at the same laboratory in fresh blood samples. The main limitations are a clinic-based report and not a population-based one, the inclusion of different ethnic groups, the lack of follow-up, and a limited reproducibility due to a single OGTT test. On the other hand, repeated OGTT in pediatric patients might raise ethical problems. Further studies, in particular a longitudinal evaluation of obese children and adolescents with normal glycometabolic assessment at the first evaluation, are desirable.

## Data Availability

Data are available upon request.
